# Use of the SAMe-TT_2_R_2_ Score to Predict Good Anticoagulation Control with Warfarin in Chinese Patients with Atrial Fibrillation: Relationship to Ischemic Stroke Incidence

**DOI:** 10.1371/journal.pone.0150674

**Published:** 2016-03-24

**Authors:** Pak Hei Chan, Jo Jo Hai, Esther W. Chan, Wen Hua Li, Hung Fat Tse, Ian C. K. Wong, Gregory Y. H. Lip, Chung Wah Siu

**Affiliations:** 1 Cardiology Division, Department of Medicine, Li Ka Shing Faculty of Medicine, the University of Hong Kong, Hong Kong SAR, China; 2 Department of Pharmacology and Pharmacy, Li Ka Shing Faculty of Medicine, the University of Hong Kong, Hong Kong SAR, China; 3 Department of Cardiovascular Ultrasound and Non-invasive Cardiology, Sichuan Academy of Medical Sciences and Sichuan Provincial People's Hospital, Chengdu, Sichuan, China; 4 University of Birmingham Centre for Cardiovascular Sciences, City Hospital, Birmingham, United Kingdom; University of Glasgow, UNITED KINGDOM

## Abstract

**Background:**

The efficacy and safety of warfarin therapy for stroke prevention in atrial fibrillation (AF) depends on the time in therapeutic range (TTR). We aimed to assess the predictive ability of SAMe-TT_2_R_2_ score in Chinese AF patients on warfarin, whose TTR is notoriously poor.

**Methods and Results:**

This is a single-centre retrospective study. Patients with non-valvular AF on warfarin diagnosed between 1997 and 2011 were stratified according to SAMe-TT_2_R_2_ score, and TTR was calculated using Rosendaal method. The predictive power of SAMe-TT_2_R_2_ scores for good TTR i.e. >70% was assessed. We included 1,428 Chinese patients (mean age 76.2±8.7 years, 47.5% male) with non-valvular AF on warfarin. The mean and median TTR were 38.2±24.4% and 38.8% (interquartile range: 17.9% and 56.2%) respectively. TTR decreased progressively with increasing SAMe-TT_2_R_2_ score (p = 0.016). When the cut-off value of SAMe-TT_2_R_2_ score was set to 2, the sensitivity and specificity to predict TTR<70% were 85.7% and 17.8%, respectively. The corresponding positive and negative predictive values were 10.1% and 92.0%. After a mean follow-up of 4.7±3.6 years, 338 patients developed an ischemic stroke (4.96%/year). Patients with TTR≥70% had a lower annual risk of ischemic stroke of 3.67%/year compared with than those with TTR<70% (5.13%/year)(p = 0.08). Patients with SAMe-TT_2_R_2_ score ≤2 had the lowest risk of annual risk of ischemic stroke (3.49%/year) compared with those with SAMe-TT_2_R_2_ score = 3 (4.56%/year), and those with SAMe-TT_2_R_2_ score ≥4 (6.41%/year)(p<0.001). There was also a non-significant trend towards more intracranial hemorrhage with increasing SAMe-TT_2_R_2_ score.

**Conclusions:**

The SAMe-TT_2_R_2_ score correlates well with TTR in Chinese AF patients, with a score >2 having high sensitivity and negative predictive values for poor TTR. Ischemic stroke risk increased progressively with increasing SAMe-TT_2_R_2_ score, consistent with poorer TTRs at high SAMe-TT_2_R_2_ scores.

## Introduction

Warfarin therapy effectively reduces ischemic stroke and mortality amongst patients with non-valvular atrial fibrillation (AF).[1–3] The efficacy and safety of warfarin, however, very much depends on the quality of anticoagulation control, as assessed by the time in therapeutic range (TTR), with the proportion of time spent within therapeutic range of 2.0–3.0.[4–7] It is generally accepted that patients on warfarin should spend more than 65%, or even 70%, of time with INR between 2–3 to obtain the benefit as well as safety of the therapy.[8, 9] In Asian countries, anticoagulation control is notoriously poor, both in real world practice and in randomized clinical trials.[10]

Recently, a new clinical score, the SAMe-TT_2_R_2_ score was derived and validated using a primarily white Caucasian population to predict the likelihood of AF patients on warfarin of having a good TTR (with SAMe-TT_2_R_2_ score 0–2).[11] Given that non-Caucasian race already confers 2 points in this score, the SAMe-TT_2_R_2_ score requires validation and/or re-calibration in a non-Caucasian population.

In this study, we aimed, for the first time, to evaluate the ability of SAMe-TT_2_R_2_ score in predicting the quality of anticoagulation control (as reflected by TTR) in an Asian population.

## Methods

### Study Design and Patients

This was a retrospective study based on a hospital-based AF registry as previously described, [2–4, 12, 13] and was approved by the Institutional Review Board of the University of Hong Kong / Hospital Authority Hong Kong West Cluster. Consent was waived as all the data were analyzed anonymously. Briefly, patients ≥18 years of age diagnosed to have AF in Queen Mary Hospital, Hong Kong, from July 1997 to December 2011, were identified via a computerized database of clinical management system. Patients were excluded from the current study if they had significant valvular heart disease (i.e. prosthetic heart valve, rheumatic heart disease), less than 10 retrievable INR measurements, or interruption of warfarin for >2 weeks. Those with incomplete data or missing follow up were excluded from this study.

### Definitions

The SAMe-TT_2_R_2_ score (**S**: Sex [female] [1 point]; **A**: age <60 years [1 point]; **Me**: Medical History [>2 of the following comorbidities: hypertension, diabetes, coronary artery disease/myocardial infarction, peripheral arterial disease, congestive heart failure, previous stroke, pulmonary disease, hepatic or renal disease] [1 [point]; **T**: Treatment [interacting drugs e.g. Amiodarone for rhythm control] [1 point]; **T**: Tobacco use (within 2 years) [2 points]; and **R**: Race [non-white] [2 points] was calculated for each individual.[11] Since all patients in our study were Chinese i.e. non-White population, the minimum score will be 2 points. Subsequent occurrence of risk factor contributory to the SAMe-TT_2_R_2_ score had not been taken into account.

In addition, ischemic stroke risk was estimated at baseline using the CHA_2_DS_2_-VASc score (**C**: congestive heart failure [1 point]; **H**: hypertension [1 point]; **A**_**2**_: age 65–74 years [1 point] and age ≥75 years [2 points]; **D**: diabetes mellitus [1 point]; **S**: prior stroke or transient ischemic attack [2 points]; **VA**: vascular disease [1 point]; and **Sc**: sex category [female] [1 point]) as described in recent guidelines.[14] Likewise, the HAS-BLED score was calculated at baseline as a measure of bleeding risk.[15] Uncontrolled hypertension was defined as systolic blood pressure >160 mmHg at baseline and subsequent visit-to-visit changes in systolic blood pressure had not been taken in account. Similarly, liver disease as determined by the derangement in liver biochemistry and renal disease as determined by serum creatinine level were only assessed at baseline, subsequent changes had not been taken into account.

According to the center’s protocol, INR was measured every 8 weeks and more frequently when INR was not within the therapeutic range. The TTR was calculated for each patient using Rosendaal method, [16] in which INR was assumed to change in a linear manner between measurements, and INR values on the days with no measurement were interpolated. However, INR measurements within the first 6 weeks of warfarin therapy were excluded from this analysis due to the more frequent INR testing and large fluctuation in measurements during initial warfarin adjustment. The percentage of time during which a patient had an INR within 2.0–3.0 was taken as the TTR. According to the European guidelines, [9] a TTR≥70% was considered the criterion for ‘good anticoagulation control’.[8]

### Statistical Analysis

Continuous and discrete variables are expressed as mean ± standard derivation and percentages, respectively. Statistical comparisons of the baseline clinical characteristics were performed using Student’s *t* test, Mann-Whitney U test, or one-way ANOVA as appropriate. Binary and linear hazards regression models were used to calculate hazard ratios (HRs) of some predictive factors and their 95% confidence interval (CIs) for poor anticoagulation control, TTR<70%. The predictive performance of the SAMe-TT_2_R_2_ score for ischemic stroke was assessed using the c-statistics (area under the curve). The c-statistic for receiver operating characteristic curve was calculated using Analyze-It for Excel with the Delong-Delong comparison for c-statistic. The c-statistic integrates measures of sensitivity and specificity of the range of a variable. Ideal prediction yields a c-statistic of 1.00 whereas a value of <0.5 indicates that the prediction is no better than chance. Calculations were performed using SPSS software (version 21.0) and MedCal (version 13.1.2). All tests were two-sided, and *p*-values were considered significant if <0.05.

## Results

**Fig 1:**
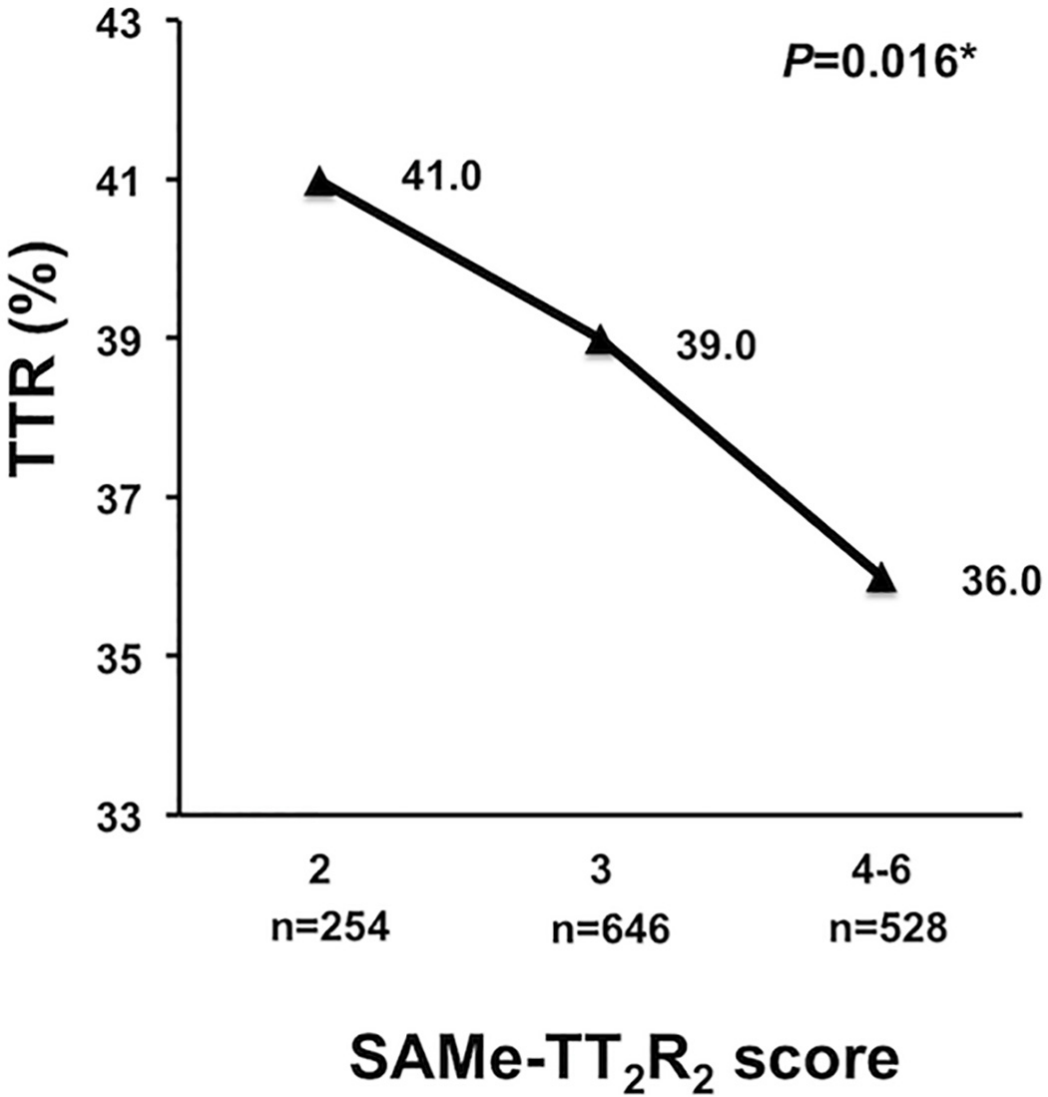
Relation between different SAMe-TT_2_R_2_ scores and the time of therapeutic range (TTR) in Chinese AF patients.

We included 1,428 Chinese patients (mean age 76.2±8.7 years, 47.5% male) with non-valvular AF on warfarin [Table 1]. The mean and median TTR overall were 38.2±24.4% and 38.8% (interquartile range: 17.9% and 56.2%) respectively. During the 14-year study period, the mean INR improved from 37.3±23.7% between 1997 and 2001 and 36.8±24.1% between 2002 and 2007, to 43.1±26.0% between 2008 and 2011. As in the original derivation cohort and subsequent external validation cohort, TTR decreased progressively with increasing SAMe-TT_2_R_2_ score (p = 0.016) (Fig 1). Even amongst patients with the lowest SAMe-TT_2_R_2_ score, i.e., 2, TTR was only 41.0±23.3%, which decreased to 39.0±25.0% and 36.0±24.0% amongst patients with SAMe-TT_2_R_2_ score of 3, and ≥4 respectively.

**Table 1:** Baseline characteristics.

	All (n = 1,428)	TTR		*p-*value^1^
		≥70% (n = 154)	<70% (n = 1,274)	
Mean age, (yrs)	76.2±8.7	73.8±9.2	76.4±8.6	<0.01*
Age<60 years, n (%)	48 (3.4)	11 (7.1)	37 (2.9)	<0.01*
Female, n (%)	671 (52.5)	79 (51.3)	671 (52.7)	0.75
HT, n (%)	922 (64.6)	102 (66.2)	820 (64.4)	0.65
DM, n (%)	387 (27.1)	32 (20.8)	355 (27.9)	0.06
Tobacco use (within 2 yrs), n (%)	71 (5.0)	11 (7.1)	60 (4.7)	0.19
Dialysis, n (%)	29 (2.0)	0 (0)	29 (2.3)	0.07
Heart failure, n (%)	367 (25.7)	335 (26.3)	32 (20.8)	0.14
CAD, n (%)	407 (28.5)	365 (28.6)	42 (27.3)	0.72
PAD, n (%)	102 (7.1)	13 (8.4)	89 (7.0)	0.51
Stroke/TIA, n (%)	496 (34.7)	68 (44.2)	428 (33.6)	<0.01*
CHA_2_DS_2_-VASc				
Mean CHA_2_DS_2_-VASc	4.2±1.6	4.1±1.5	4.2±1.6	0.34
Median CHA_2_DS_2_-VASc (IQR)	4 (3, 5)	4 (3, 5)	4 (3, 5)	0.42
Mean HAS-BLED	2.3±0.9	2.3±0.9	2.3±0.9	0.83
Treatment with interacting drugs	94 (6.6)	10 (6.5)	84 (6.6)	0.96
SAMe-TT_2_R_2_ score				0.02*
2, n (%)	254 (17.8)	22 (14.3)	232 (18.2)	
3, n (%)	646 (45.2)	80 (51.9)	566 (44.4)	
4, n (%)	442 (31.0)	41 (26.6)	401 (31.5)	
5, n (%)	75 (5.3)	7 (4.5)	68 (5.3)	
6, n (%)	11 (0.8)	4 (2.6)	7 (0.5)	

Abbreviations: CAD: Coronary artery disease; DM: diabetes mellitus; IQR: interquartile range; PAD: peripheral arterial disease; TIA: transient ischemic attack.

^1^*p-*value for comparison between patients with TTR ≥70% and TTR<70%.

* *p*-value <0.05

In the whole cohort, only 154 patients (10.7%) had good anticoagulation control (i.e., TTR≥70%) and were younger (73.8±9.2 years vs. 76.4±8.6 years, *p<*0.01), had a higher proportion with age <60 years (7.1% vs. 2.9%, *p*<0.01), and previous stroke/transient ischemic stroke (44.2% vs. 33.6%, *p<*0.01), compared with those with TTR<70%. Table 2 summarizes the HRs and the corresponding 95% CIs of baseline characteristics to poor TTR (TTR<70%) in both univariate and multivariate analysis. Of note, diabetes mellitus and heart failure were independently associated with TTR <70%, whereas age less than 60 years, previous stroke or transient ischemic attack, and lower SAMe-TT_2_R_2_ score appeared to be associated with good TTR (Table 2).

**Table 2:** Association between baseline factors and poor time in therapeutic range (TTR) <70%).

	Univariate analysis		Multivariate analysis	
	HR (95% CI)	*p*-value	HR (95% CI)	*p*-value
Age<60 years	0.39 (0.19–0.80)	0.008*	0.39 (0.18–0.84)	0.016*
Female	1.06 (0.76–1.48)	0.75		
HT	0.92 (0.64–1.31)	0.65		
DM	1.47 (0.98–2.21)	0.06	1.57 (1.02–2.41)	0.04*
Tobacco use within 2 yrs	0.64 (0.33–1.25)	0.19		
Heart failure	1.36 (0.90–2.05)	0.14	1.33 (0.87–2.04)	0.19
CAD	1.07 (0.74–1.56)	0.72		
PAD	0.82 (0.44–1.50)	0.51		
Stroke/TIA	0.64 (0.46–0.90)	0.01*	0.64 (0.45–0.90)	0.01*
CHA_2_DS_2_-VASc	1.05 (0.95–1.17)	0.34		
HAS-BLED	1.02 (0.84–1.24)	0.83		
Treatment with interacting drugs	1.02 (0.52–2.00)	0.96		
SAMe-TT_2_R_2_ score		0.037*		0.15
2, n (%)	Reference		Reference	
3, n (%)	0.67 (0.41–1.10)	0.12	0.58 (0.35–0.96)	0.04*
4, n (%)	0.93 (0.54–1.60)	0.79	0.77 (0.43–1.38)	0.39
≥ 5, n (%)	0.65 (0.30–1.40)	0.27	0.67 (0.28–1.60)	0.47

* *p*-value <0.05

When the cutoff value of SAMe-TT_2_R_2_ score was set to 2, the sensitivity and specificity to predict TTR≥70% were 85.7% and 18.2%, respectively. The corresponding positive and negative predictive values were 11.2% and 91.3%. The Youden index was 0.039. If the cutoff value to predict TTR≥70% was set to 3, the sensitivity and specificity to detect TTR≥70% were lower, being 66.2% and 37.4%, respectively; however, the positive and negative predictive values were similar, being 11.8% and 89.7%, respectively. The Youden index for the cutoff of 3 was 0.036.

**Fig 2:**
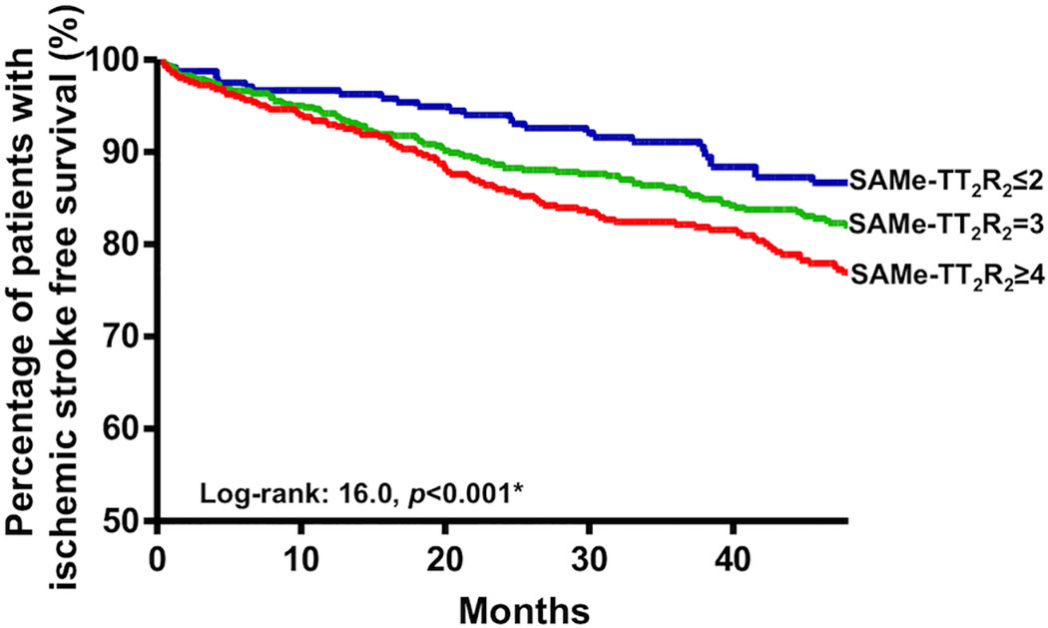
Kaplan-Meier estimates of ischemic stroke-free survival in Chinese AF patients with different SAMe-TT_2_R_2_ scores.

**Fig 3:**
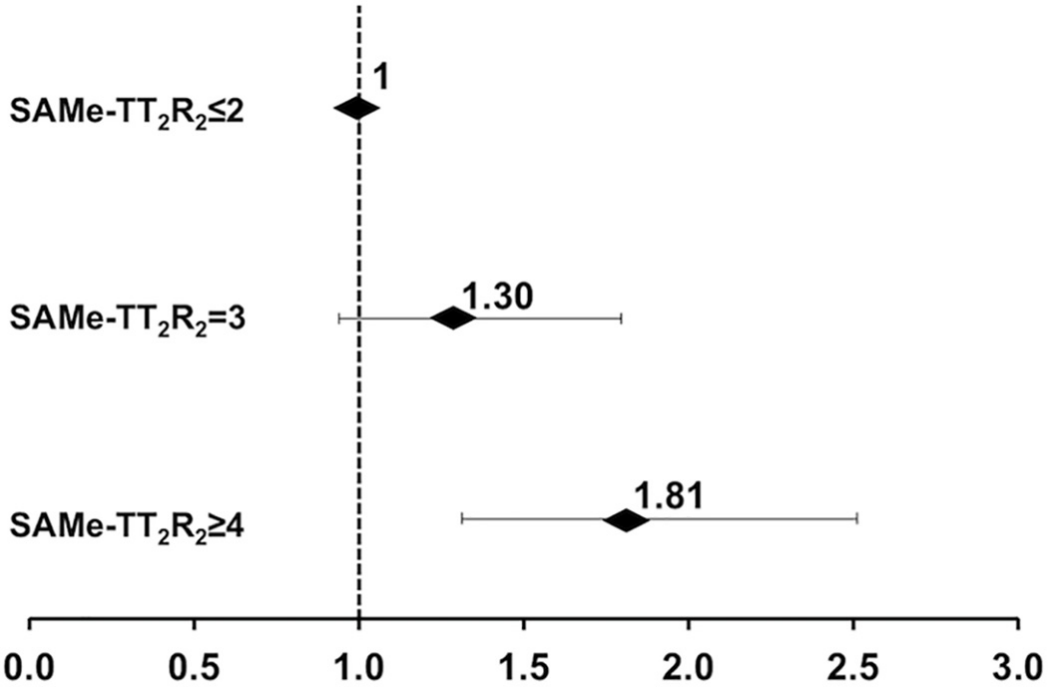
Hazard Ratios of different strata of SAMe-TT_2_R_2_ scores on ischemic stroke. Horizontal lines represent 95% confident intervals (CIs) around point estimates.

After a mean follow-up of 4.7±3.6 years, 338 patients developed an ischemic stroke with an annual incidence of 4.96%/year. Patients with TTR≥70% had a lower annual risk of ischemic stroke of 3.67%/year compared with than those with TTR<70% 5.13%/year (*p* = 0.08). The SAMe-TT_2_R_2_ score showed a significant association with annual risk of ischemic stroke. Fig 2 shows a Kaplan Meier analysis of ischemic stroke amongst patients with different strata of SAMe-TT_2_R_2_ score (Log-rank: 16.0, *P*<0.001). Patients with SAMe-TT_2_R_2_ score ≤2 had the lowest risk of annual risk of ischemic stroke (3.49%/year) compared with those with SAMe-TT_2_R_2_ score = 3 (4.56%/year), and those with SAMe-TT_2_R_2_ score ≥4 (6.41%/year)(*p<*0.001). Fig 3 summarizes HR and the corresponding 95% CIs of different strata of SAMe-TT_2_R_2_ score for ischemic stroke. At follow up, there were altogether 63 intracranial haemorrhages with annual incidence of 0.90%/year. There was a non-significant trend towards more events with increasing SAMe-TT_2_R_2_ score, with events rates at scores 2, 3 and ≥4 being 0.77%/year, 0.96%/year (HR: 1.23, 95% CI: 0.62–2.45), and 0.90%/year (HR: 1.09, 95% CI: 0.52–2.26), respectively. However, the area under the curve of the SAMe-TT_2_R_2_ score for stroke prediction was only 0.543 (95% CI: 0.52–0.57) with the Youden index of 0.08.

## Discussion

In this study, we have shown for the first time that amongst Chinese AF patients on warfarin, the SAMe-TT_2_R_2_ correlated with TTR in Chinese AF patients, with a SAMe-TT_2_R_2_ score >2 having a high sensitivity and negative predictive value for good TTR. Second, recalibration of the score in this non-Caucasian population did not improve its sensitivity. Third, the incidence of ischemic stroke increased progressively with increasing SAMe-TT_2_R_2_ score, consistent with poor TTRs at high SAMe-TT_2_R_2_ scores.

The importance of good quality anticoagulation control amongst patients on warfarin therapy typically with a TTR above 65% to 70% cannot be emphasized. In Asian population including Chinese, quality of anticoagulation has generally been poor [10], with a median TTR amongst Chinese AF patients being as low as 38.8% in the present cohort. Poor TTR undermines not only the efficacy of the therapy (more ischemic stroke), but also the safety (more intracranial bleeding).[4] Indeed, this might partly explain the prevailing perception of warfarin as an ineffective-and-yet-dangerous drug amongst Chinese clinicians, leading to gross underutilization of the therapy in Chinese and resulting is major missed opportunities for stroke prevention.

The SAMe-TT_2_R_2_ score, which was initially derived and validated in the white Caucasian population, facilitates decision making by clinicians to help predict the likelihood of achieving good quality anticoagulation control following the initiation of warfarin therapy.[11] In general, patients are expected to have good TTR when the SAMe-TT_2_R_2_ score is 0–2, and are at risk of suboptimal anticoagulation control when the SAMe-TT_2_R_2_ score >2. Rather than imposing a ‘trial of warfarin’ to see if high TTRs can be achieved, and putting such inception cohort patients at risk of suboptimal INRs and increased stroke risk (by 70%)[17], those patients with SAMe-TT_2_R_2_ score >2 could be targeted upfront for better follow up and educational efforts [18], or alternative anticoagulant strategies (e.g. non-vitamin K antagonist oral anticoagulants (NOACs)). Given the overall poor TTR in the Chinese AF population, NOACs should perhaps be considered as the first line antithrombotic agents for stroke prevention in non-valvular AF; warfarin therapy might be considered only when NOAC is contraindicated as in patients with end-stage renal disease.

Similar to the original derivation cohort, [11] an increase in the SAMe-TT_2_R_2_ score likewise resulted in a decrease in TTR in our cohort of Chinese AF patients and as many other non-Asian cohorts have shown, higher stroke rates [6, 19]. For the same SAMe-TT_2_R_2_ score, Chinese AF patients still appear to have a poorer TTR compared with white Caucasians [20], implying that other strategies (e.g. NOACs) may be better options. As rightly predicted by the score, around 90% of patients in the present cohort with the SAMe-TT_2_R_2_ score ≥2 did not have good anticoagulation control. This is consistent with epidemiological and trial data showing that Asians seem to do poorly on warfarin with higher risks of thromboembolism and bleeding (particularly intracranial haemorrahge)[10]. Albeit qualitatively consistent, deviating the default cutoff value of 2 from the original derivation cohort would still make the score less useful in Chinese AF population, with a lowered sensitivity and negative predictive value by such recalibration.

Although the inclusion of an ethnicity component into the SAMe-TT_2_R_2_ score appears to a practical way to improve the predictive power of the score, this may oversimplify the observed difference in TTR down to ethnicity *per se*. The reasons underlying poor TTR may be highly population-specific, ranging from genetic, dietary, behavioural to even health care provision system. Individual factors constituting the SAMe-TT_2_R_2_ score may affect the TTR in different ways in different ethnic groups with different life-style, value and belief. Most obviously, in the primarily Caucasian population, younger age represents a risk factor for poor TTR whereas in Hong Kong Chinese, younger age may in fact be associated with better understanding to diet restriction and importance of TTR, which may then in turn translate into a better TTR. Likewise in primarily Caucasian population, previous stroke was somehow associated with poor TTR, but amongst Chinese in Hong Kong, patients with previously stroke had a better TTR, which may reflect a more compliant lifestyle after stroke. Unfortunately, we lack a parallel Caucasian cohort in Hong Kong for comparison. For the SAMe-TT_2_R_2_ score, being Chinese is undoubtedly associated with poor TTR but higher SAMe-TT_2_R_2_ score may be the result of a combination of certain protective factors and risk factors for poor TTR, thus may contribute to either better or poorer TTR. Additional cultural-specific factors such as the frequency of traditional Chinese medicine use may be necessary to further improve the performance of the SAMe-TT_2_R_2_ score, but this could be remedied by extending the T criterion of the SAMe-TT_2_R_2_ score for ‘Treatment [interacting drugs]’ to include ‘Treatment [interacting drugs] or high intake of diet/foods that interfere with warfarin’. Some of the clinical factors within the SAMe-TT_2_R_2_ score are also risk factors for stroke, but the SAMe-TT_2_R_2_ score remains a simple validated score that has been shown to predict labile INRs, thromboembolism, death and serious bleeding events [19].

### Limitations

This study is limited by its registry-based and single-centre observational design in primarily hospital-based patients. The variance in the management including the overall quality and facilities offered to patients for anticoagulation control might differ over the study period of 14 years. Information about the frequency and magnitude of warfarin dose changes during follow up was not recorded in this cohort. We were also suboptimally powered for serious bleeding events, including intracranial haemorrhage. The choice of target TTR of 70% instead of 65% as suggested by the NICE guideline in the UK is somewhat arbitrary. However, even the target TTR is to be lowered to 65%, the percentage of patients achieving the target TTR remains very small (14.8%).

## Conclusion

The SAMe-TT_2_R_2_ score correlates well with TTR in Chinese AF patients, with a score >2 having high sensitivity and negative predictive values for poor TTR. The risk of ischemic stroke increased progressively with increasing SAMe-TT_2_R_2_ score, consistent with poorer TTRs at high SAMe-TT_2_R_2_ scores.
